# BRD4 regulates cellular senescence in gastric cancer cells via E2F/miR-106b/p21 axis

**DOI:** 10.1038/s41419-017-0181-6

**Published:** 2018-02-12

**Authors:** Xingchen Dong, Xiangming Hu, Jinjing Chen, Dan Hu, Lin-Feng Chen

**Affiliations:** 1 Department of Biochemistry, University of Illinois at Urbana-Champaign, Urbana, IL 61801 USA; 2 Department of Pathology, Fujian Provincial Cancer Hospital, The Affiliated Hospital of Fujian Medical University, Fujian, China 350108; 3 Institute for Translational Medicine, School of Basic Medical Sciences, Fujian Medical University, Fuzhou, Fujian, China 350108; 4 Department of Medical Biochemistry, College of Medicine, University of Illinois at Urbana-Champaign, Urbana, IL 61801 USA

## Abstract

Small molecules targeting bromodomains of BET proteins possess strong anti-tumor activities and have emerged as potential therapeutics for cancer. However, the underlying mechanisms for the anti-proliferative activity of these inhibitors are still not fully characterized. In this study, we demonstrated that BET inhibitor JQ1 suppressed the proliferation and invasiveness of gastric cancer cells by inducing cellular senescence. Depletion of BRD4, which was overexpressed in gastric cancer tissues, but not other BET proteins recapitulated JQ1-induced cellular senescence with increased cellular SA-β-Gal activity and elevated p21 levels. In addition, we showed that the levels of p21 were regulated at the post-transcriptional level by BRD4-dependent expression of miR-106b-5p, which targets the 3′-UTR of *p21* mRNA. Overexpression of miR-106b-5p prevented JQ1-induced p21 expression and BRD4 inhibition-associated cellular senescence, whereas miR-106b-5p inhibitor up-regulated p21 and induced cellular senescence. Finally, we demonstrated that inhibition of E2F suppressed the binding of BRD4 to the promoter of miR-106b-5p and inhibited its transcription, leading to the increased p21 levels and cellular senescence in gastric cancer cells. Our results reveal a novel mechanism by which BRD4 regulates cancer cell proliferation by modulating the cellular senescence through E2F/miR-106b-5p/p21 axis and provide new insights into using BET inhibitors as potential anticancer drugs.

## Introduction

Epigenetic regulation of gene expression plays important roles in controlling normal cellular functions as well as abnormal cellular activities in human diseases like cancer. Three different types of proteins are involved in the epigenetic regulation: enzymes that modify histone or DNA (writers), enzymes that remove modifications on histone or DNA (erasers), and proteins that recognize these modifications (readers)^1^. By changing the modifications on histones and DNA, the epigenetic regulators alter the non-covalent interactions within and between nucleosomes, leading to altered chromatin structures and gene expression^1^. Aberrant expression patterns and genomic alterations of epigenetic regulators are found in a variety of cancers, highlighting the importance of epigenetic regulation of gene expression in tumorigenesis^2^. Drugs targeting epigenetic regulators have emerged as novel therapies in cancer treatment.

The bromodomain-containing proteins represent a class of epigenetic readers that recognize acetylated lysines of histone and non-histone proteins via their bromodomains^3^. BRD4, one of the BET (bromodomain and extra-terminal) family proteins, has become a key player in transcription, cell cycle control, inflammatory cytokine production and cancer development^4,5^. BRD4 is involved in the development of hematological malignancies and solid tumors, emerging as a promising therapeutic target for cancer treatment^6^. Small molecules targeting bromodomains of BRD4 and other BET family proteins display strong anti-tumor activities, suppressing the proliferation and transformation potential of various cancer cells^7–9^. These BET inhibitors (BETis) bind to the acetylated lysine recognition pocket within bromodomains and competitively block the binding of BET family bromodomains to histones or non-histone proteins^7,10,11^. BETis suppress cancer cell proliferation via distinct mechanisms, including cell apoptosis, cell cycle arrest, and cellular senescence^12^. However, the exact contribution of each BET protein and the underlying mechanisms in BETi-mediated inhibition of cancer cell proliferation are not fully characterized.

Cellular senescence is a state by which cells adopt a permanent, irreversible cell cycle arrest and cease to divide^13^. Cellular senescence is triggered when cells sense various stresses, including shortening of telomeres, activation of oncogenes and inactivation of tumor suppressors, and DNA damage^14^. Inhibition of epigenetic regulators also induces cellular senescence^15^. Cellular senescence is usually accompanied by morphological changes with enlarged and flatted cell shape, increased senescence-associated β-galactosidase (SA-β-Gal) activity, and changed levels of cell cycle related proteins^14^. Up-regulation of cellular levels of cyclin-dependent kinase inhibitor p21 (also known as p21^WAF1/Cip1^ or CDKN1) has been implicated in cellular senescence and represents one of the hallmarks of senescence^14^. The expression of p21 is regulated at multiple levels, including transcriptional, post-transcriptional, and post-translational levels^16^. At the transcriptional level, the expression of p21 can be regulated by p53^16,17^. p53 directly binds to the promoter region of p21 and activates its transcription in response to DNA damage and cell cycle arrest^14,17^. The expression of p21 is also regulated in a p53-independent manner at the post-translational level^16^. For example, the cellular levels of p21 were regulated by SCF^Skp2^-mediated ubiquitination and degradation^18,19^. At the post-transcriptional level, the expression of p21 can be regulated by noncoding RNAs, especially microRNAs^20^.

MicroRNAs (miRNAs) are small, endogenous noncoding RNAs of 18–24 nucleotides in length and play significant roles in numerous cellular processes, including cell cycle arrest, cell proliferation and death, and cellular senescence^21^. miRNAs bind to the 3′ untranslated region (3′-UTR) of target mRNAs via nucleotide pairing between nucleotides 2 to 7 of the miRNA and the corresponding sequence of the target 3′-UTR, decreasing the mRNA stability, translation and the production of target proteins^22^. Aberrantly expressed miRNAs are identified in many cancers and have been shown to associate with tumor development, progression and response to cancer therapy^23^. The transcription of miRNAs is carried out by RNA polymerase II (RNAPII) and is regulated by RNAPII-associated transcription factors and epigenetic regulators^24^. Most of the miRNAs are encoded by introns of non-coding or coding transcripts^24^. The intronic miRNAs often share the same regulatory units of the host genes and are transcribed in the same direction in coordination with the pre**-**messenger RNA in which they reside^24^. BRD4 is known to regulate RNAPII-dependent gene expression^5^, however, it is largely undetermined whether BRD4 regulates miRNA transcription in cancer cells and whether BRD4-regulated miRNAs contribute to BDR4-dependent cancer cell proliferation.

In an effort to understand the contribution of BET to gastric cancer development and the mechanism for BETi-mediated inhibition of cancer cell proliferation, we found that BRD4 was overexpressed in gastric cancer patient tissues and BET inhibitor JQ1 targeted BRD4 to induce cellular senescence in gastric cancer cells. BRD4 was recruited to the promoter of miR-106b-5p via E2F and facilitated the transcription of miR-106b-5p, which in turn targets 3′-UTR of p21 to regulate cellular senescence.

## Results

### JQ1 inhibits the proliferation and invasiveness of gastric cancer cells

**Fig. 1:**
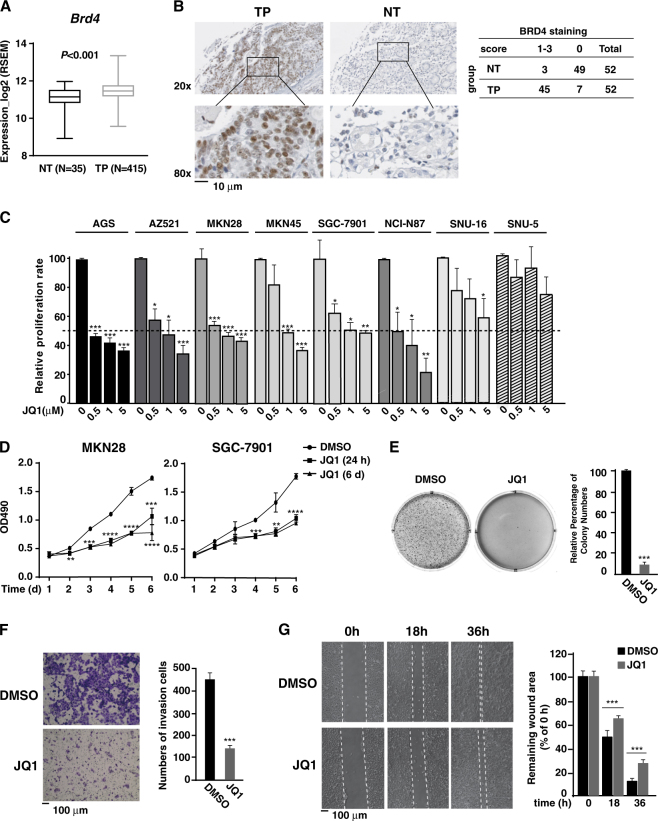
JQ1 inhibits the proliferation, migration, and invasion of gastric cancer cell lines. **a** Box-plots of BRD4 mRNA levels in normal tissue (NT, patient number = 35) vs primary of tumor (TP, patient number = 415) acquired from TCGA Firebrowse portal are visualized using GraphPad Prism, and the *p*-value is computed and displayed. **b** Left: Representative of immunohistochemical (IHC) staining of BRD4 in human NT vs TP tissues. Boxed regions are enlarged to the bottom of each image. Right: IHC staining score summary from 52 gastric cancer samples and the paired normal tissue samples is shown in the table on the right. The Pearson’s chi-square test (*χ*^2^ = 68.250, *p* < 0.001) is utilized to evaluate the likelihood of the different expression levels of BRD4 in NT vs TP samples. **c** Various gastric cancer cells were treated with JQ1 of indicated concentration for 72 h, and cell proliferation was measured by A490 nm using the CellTiter 96^R^AQueous One Solution cell proliferation assay (MTS) (Promega). Data represent the mean of three independent experiments. Dot line represents the 50% of growth inhibition. **d** MKN28 or SGC-7901 cells were treated with DMSO or 5 μM of JQ1 for 24 h or 6 days, and cell proliferation was measured at different time points as in **c**. **e** MKN28 cells were seeded in soft-agar and cultured for 15 days with DMSO or 5 μM of JQ1. Representative photographs were taken at day 21. **f** MKN28 cells were treated with DMSO or 5 μM of JQ1, and cell invasion assay was performed using Transwell invasion chambers (Becton, Dickinson, and Company) according to manufacturer’s instructions. **g** The wound-healing migration assays for MKN28 cells in the presence of DMSO or 5 μM of JQ1. Representative photographs were taken at 0, 18, and 36 h (left). The percentage of the average speed of wound closure from three independent experiments ± SD is shown on the right

To explore the potential therapeutic effect of BETis on gastric cancer, we first compared the mRNA levels of BETs in gastric cancer patients and normal gastric tissues using data acquired through TCGA Firebrowse portal (http://firebrowse.org/). The mRNA levels of BRD3 and BRD4 were significantly higher in primary of tumor (TP) versus normal tissue (NT) (Fig. 1a and Fig. S1), while there was no significant change of BRD2 mRNA levels in NT vs. TP (Fig. S1). Consistently, in a tissue array with 52 TP and 52 paired NT samples, we found that almost 87% of TP samples (45 out of 52) displayed higher protein levels of BRD4 while 94% of NT samples (49 out of 52) showed low protein levels BRD4 (Fig. 1b). Statistical analysis reveals that BRD4 is highly overexpressed in gastric cancer tissues (Fig. 1b), suggesting that BRD4 might contribute to the proliferation of gastric cancer cells.

We next examined the effect of JQ1, a pan-BET inhibitor, on cell proliferation of a panel of gastric cancer cell lines. While the effect of JQ1 on the proliferation of cells varied, all the tested gastric cancer cells showed a certain degree of growth inhibition by JQ1 in a dose-dependent manner (Fig. 1c). AGS, AZ521, MKN28, SGC-7901, NCI-N87, and MKN45 cells were more sensitive to JQ1 than SNU-16 and SNU-5 cells (Fig. 1c). Continuous treatment of MKN28 and SGC-7901 cells with 5 μM of JQ1 for a longer period of time (6 days) efficiently blocked the gastric cancer cell proliferation (Fig. 1d). Interestingly, treatment of gastric cancer cells with 5 μM of JQ1 for only 24 h was also sufficient to inhibit the cell proliferation (Fig. 1d), indicating an irreversible inhibitory effect of JQ1 on the proliferation of gastric cancer cells.

We next investigated the effect of JQ1 on the anchorage-independent cell growth of MKN28 cells. In the presence of JQ1, the numbers of colony formed in the soft-agar were largely reduced (Fig. 1e). The invasive potential of MKN28 cells was also inhibited by JQ1 as revealed by the Matrigel invasion assay (Fig. 1f). Furthermore, in a wound-healing assay to measure the effect of JQ1 on cell migration, we found that the scratch wound closed more slowly in JQ1-treated MKN28 cells than control cells (Fig. 1g).

### JQ1 inhibits gastric cancer cell proliferation by inducing cellular senescence

**Fig. 2:**
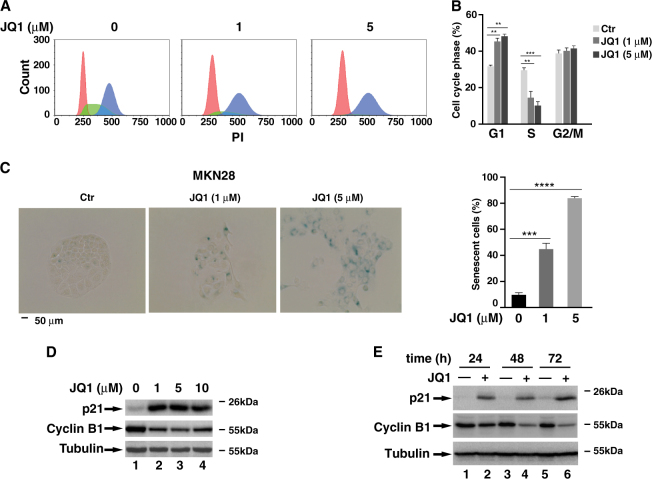
**JQ1 up-regulates p21 protein level and induces cellular senescence**. **a** Cell cycle profile of MKN28 cells after treatment with DMSO or JQ1 (1 μM or 5 μM) for 24 h. **b** The percentage of cells in different phases of cell cycle from (A) was indicated. Data represent the average of three independent experiments. **c** A total of 5 × 10^3^ MKN28 cells were treated with indicated concentration of JQ1 for 72 h, and cellular senescence was measured using Senescence β-Galactosidase Staining Kit (cell signaling). The percentage of β-Gal staining-positive cells is shown on the right. Data represent the average of three independent experiments. (D & E) MKN28 cells were treated with different concentrations of JQ1 for 24 h **d** or with 5 μM of JQ1 for different time points **e**. The cell lysates were immunoblotted for the indicated proteins

Apoptosis has been implicated as one of the mechanisms for the inhibition of cell proliferation by BETis^12^. Intriguingly, FACS analysis with Annexin V and propidium iodide (PI) staining revealed that 5 μM of JQ1, which was sufficient to suppress cell proliferation (Fig. 1d), didn’t induce apoptosis (Fig. S2). Immunoblotting for PARP also showed no significant cleavage of PARP with JQ1 treatment (Fig. S3). We also examined the cell cycle profile of these JQ1-treated cells and found that numbers of S phase cells reduced with an increased number of G1 phase cells (Figs. 2a, b). However, the numbers of G2 phase cells remained unchanged (Fig. 2a, b). These data suggest that the reduced cell proliferation of MKN28 cells likely results from JQ1-induced G1/S arrest but not from cell apoptosis or G2/M cell cycle arrest.

In addition to apoptosis, stressed cells could adopt a permanent and irreversible cell cycle arrest to undergo cellular senescence^14^. Due to the irreversible inhibitory effect of JQ1 on the proliferation of MKN28 cells (Fig. 1d), we next investigated the effect of JQ1 on cellular senescence. When MKN28 cells treated with two different doses of JQ1 for 3 days and the activity of SA-β-Gal was measured, we observed a dose-dependent increased number of SA-β-Gal-positive cells with enlarged and flattened shape (Fig. 2c). Half of the cells were SA-β-Gal-positive when treated with 1 μM of JQ1 and SA-β-Gal-positive cell number increased to ~80% when treated with 5 μM of JQ1 (Fig. 2c). A similar dose-dependent increased number of SA-β-Gal-positive cells was observed in SGC-7901, AGS, and MKN45 cells (Fig. S4). Consistently, the expression of p21, a senescence marker, was dramatically induced by different doses of JQ1 while the levels of Cyclin B1 were down-regulated in JQ1-treated MKN28 or SGC-7901 cells (Figs. 2d, e and Fig. S5).

### Inhibition of BRD4 promotes cellular senescence of gastric cancer cells

**Fig. 3:**
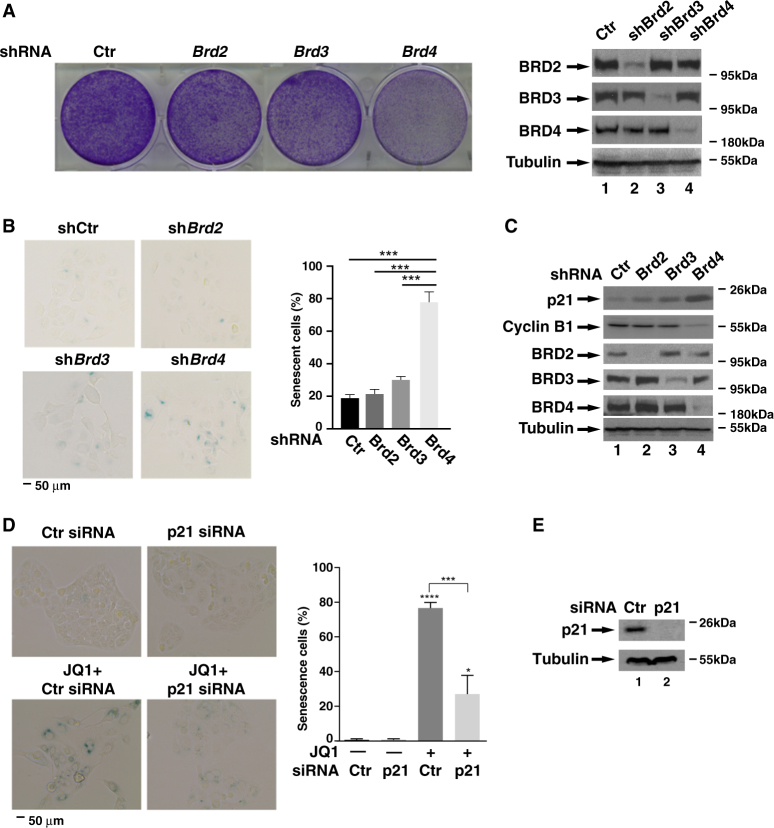
**BRD4 is involved in JQ1-induced cellular senescence**. **a** MKN28 cells were infected with lentiviruses expressing shRNAs against *Brd2*, *Brd3*, and *Brd4*, respectively. Cell proliferation was measured by clonogenic assay after 3 days. Knockdown efficiency is shown on the right panels. **b** MKN28 cells infected with lentiviruses expressing indicated shRNAs were subject to β-Gal staining as described in Fig. 2C. Percentage of β-Gal staining-positive cells is shown on the right. **c** MKN28 cells infected with lentiviruses expressing indicated shRNAs were lysed and subject to immunoblotting for indicated proteins. **d&e** MKN28 cells were transfected with either control or p21 siRNA. Twenty hours later, cells were treated with DMSO or 5 μM of JQ1 for another 3 days. β-Gal staining was performed as in Fig. 2C. Percentage of β-Gal staining-positive cells is shown on the right. Data represent the average of three independent experiments. p21 siRNA knockdown efficiency is shown in (**e**)

To determine which BET protein was involved in JQ1-induced cellular senescence, we infected MKN28 cells with lentiviruses expressing shRNA against each BET protein and evaluated the effect of these knockdowns on the proliferation of MKN28 cells via the clonogenic assay. Among three BET proteins, depletion of *Brd4* significantly reduced the number of viable cells, whereas depletion of *Brd2* or *Brd3* had little effect (Fig. 3a). These data suggest that BRD4 is the primary target in JQ1-induced proliferation inhibition and likely cellular senescence of MKN28 cells. Supporting this notion, depletion of *Brd4*, but not *Brd2* and *Brd3*, increased the number of SA-β-Gal-positive cells (Fig. 3b). In line with increased SA-β-Gal activity, the levels of p21 were enhanced in *Brd4* but not in *Brd2* and *Brd3* knockdown cells (Fig. 3c). Taken together, these results demonstrate that BRD4 regulates gastric cancer cell proliferation via cellular senescence.

p21, being a senescence marker, also directly regulates cellular senescence^25^. Depletion of *p21* by siRNA reduced the number of the SA-β-Gal-positive cells in JQ1-treated MKN28 cells (Fig. 3d, e), indicating that JQ1-induced expression of *p21* directly regulates the senescence of MKN28 gastric cancer cells.

### BRD4 regulates the 3′-UTR of *p21.* mRNA

**Fig. 4:**
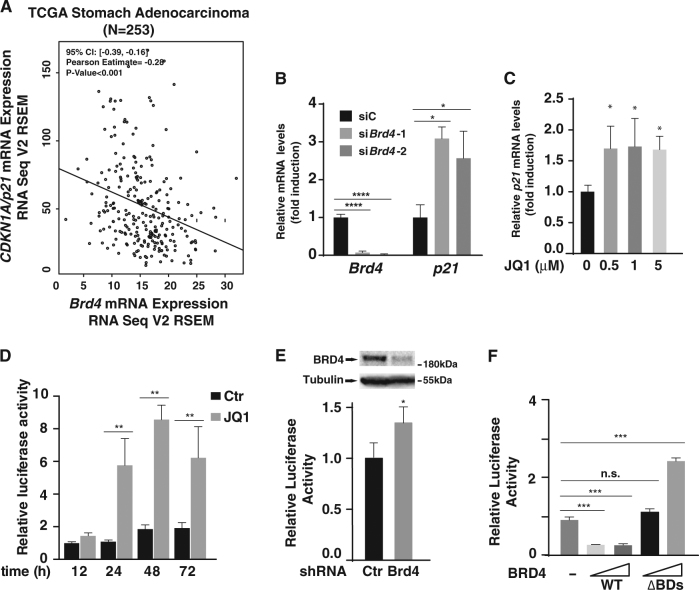
BRD4 regulates the 3′-UTR of *p21.* **a** Scatterplots of *Brd4* mRNA expression level versus *p21*/*CDKN1A* mRNA expression level in Stomach Adenocarcinoma (STAD) in The Cancer Genome Atlas (TCGA). Data are acquired from TCGA cBioportal and analyzed by R programming. 95% Confident Intervals (CI), Pearson correlation coefficients and *P* values are displayed. **b** MKN28 cells were transfected with control siRNA, and two sets of BRD4 siRNA for 24 h and the levels of *Brd4* and *p21* mRNA were measured by RT-PCR. Data represent the average of three independent experiments. **c** MKN28 cells were treated with DMSO or different concentrations of JQ1 for 24 h and the levels of *p21* mRNA were measured by RT-PCR. Data represent the average of three independent experiments. **d** The *p21* 3′-UTR luciferase reporter plasmids were transfected into MKN28 cells with or without JQ1 treatment. Luciferase activity was measured as indicated time points after transfection. Data represent the average of three independent experiments. **e** MKN28 cells were infected with lentiviruses expressing control or Brd4 shRNA. 24 h later, infected cells were transfected with *p21* 3′-UTR luciferase reporter plasmids. Luciferase activity was measured 24 h after transfection. Data represent the average of three independent experiments. **f** MKN28 cells were transfected with *p21* 3′-UTR luciferase reporter plasmids together with expression vectors for BRD4 or BRD4(ΔBDs). Luciferase activity was measured 48 h after transfection. Data represent the average of three independent experiments

Since BRD4 is a key regulator of RNAPII-dependent gene expression^5^, the dramatically enhanced cellular levels of p21 in JQ1-treated or *Brd4* knockdown MKN28 cells raised the possibility that BRD4 might regulate the transcription of *p21*. Since BRD4 is overexpressed in gastric cancer tissues (Fig. 1b)^26^, we then examined the mRNA levels of *Brd4* and *p21* (also called *CDKN1A*) in gastric cancer samples using the RNAseq datasets from TCGA (http://cancergenome.nih.gov/). Analysis of the datasets of stomach adenocarcinoma^27^ revealed that the mRNA levels of *Brd4* inversely correlated with the levels of *p21* (Fig. 4a), indicating that BRD4 might be a negative regulator of p21. To confirm this, we examined the effect of BRD4 inhibition on the expression of *p21* in MKN28 cells. Depletion of *Brd4* or treatment of MKN28 cells with JQ1 up-regulated *p21* mRNA levels with 2–3 folds induction in *Brd4* knockdown cells (Fig. 4b) and less than 2 folds induction in JQ1-treated cells (Fig. 4c). These moderately increased *p21* mRNA levels indicate that additional mechanism might be utilized by BRD4 to regulate the expression of *p21*.

In addition to the regulation at the transcriptional level, the expression of p21 can be regulated at the post-transcriptional level via 3′-UTR of *p21* mRNA by miRNAs^16,20^. We next examined whether JQ1 could affect 3′-UTR activity of *p21* mRNA. When MKN28 cells transiently transfected with *p21* 3′-UTR-luciferase plasmids were treated with JQ1, the luciferase activity was increased by JQ1 as early as 24 h (Fig. 4d). Consistently, depletion of *Brd4* also increased the activity of 3′-UTR of *p21* reporter (Fig. 4e). In contrast, overexpression of BRD4 in MKN28 cells decreased the luciferase activity of *p21* 3′-UTR luciferase reporter (Fig. 4f). The ability of BRD4 to suppress the 3′-UTR of *p21* relied on its two bromodomains since BRD4 mutant with a deletion of both bromodomains failed to suppress 3′-UTR luciferase reporter (Fig. 4f). Collectively, these data indicate that BRD4 regulates the 3′-UTR activity of *p21* mRNA likely by binding to acetylated histone or non-histone proteins via its two bromodomains.

### miR-106b-5p targets p21 to regulate the cellular senescence in BRD4-inhibited cells

**Fig. 5:**
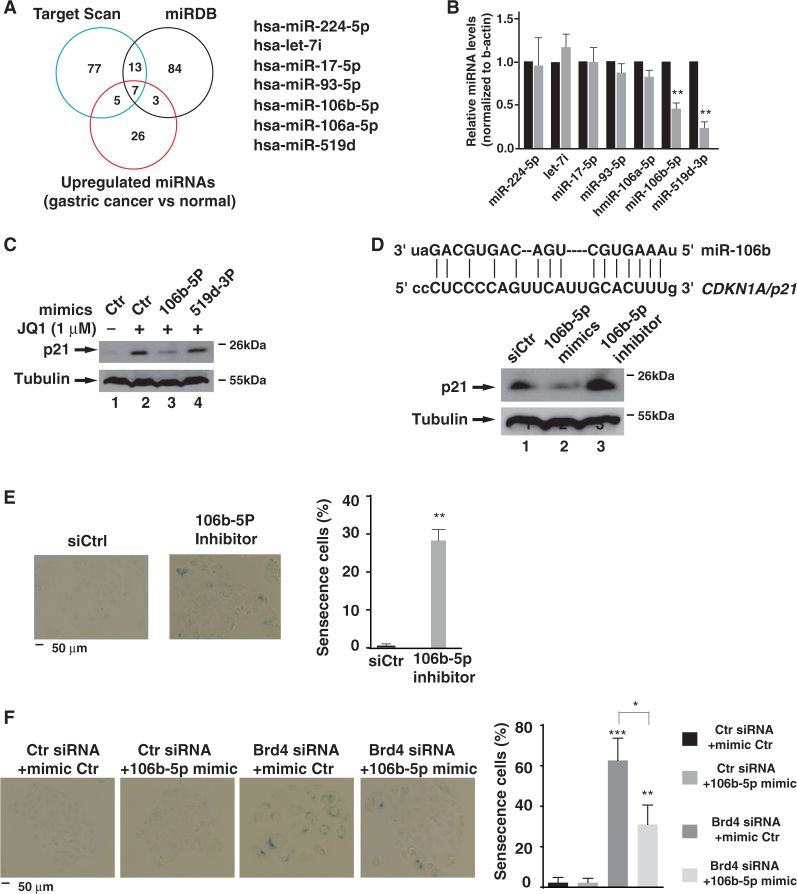
miR-106b-5p regulates cellular senescence by targeting p21. **a** Schema of the miRNA candidates targeting *p21* mRNA. The top two circles represent the number of predicted p21 miRNAs using prediction algorithms Target Scan (left) and miRDB (right). The bottom circle represents the number of miRNAs that are up-regulated in gastric cancer tissues compared to normal tissues. **b** MKN28 cells were transfected with control or BRD4 siRNA for 24 h. The levels of indicated miRNAs were measured using QuantiMir Kit (System Bioscience). Samples are from si*BRD4-1* in Fig. 4B. **c** MKN28 cells transfected with indicated miRNA mimics for 24 h were treated with JQ1 for 24 h and the levels of p21 were measured by immunoblotting with anti-p21 antibody. **d** Sequence complementarity between miR-106b-5p and 3′-UTR of *p21* mRNA (top panel). miR-106b-5p mimics and inhibitors were transfected into MKN28 cells. Forty-eight hours later, the levels of indicated proteins were measured by immunoblotting (bottom panel). **e** MKN28 cells were transfected with miR-106b-5p inhibitors for 5 days, and cells were subject to β-Gal staining (left panel). The percentage of β-Gal staining-positive cells is indicated in the right. **f** MKN28 cells were transfected with different combinations of BRD4 siRNA and miR-106b-5p mimics as indicated. After 5 days, cells were subject to β-Gal staining. The percentage of β-Gal staining-positive cells is indicated on the right

miRNAs often target 3′-UTRs of mRNAs to regulate the stability and translation efficiency of mRNAs^22^. We next explored the possibility that BRD4 might regulate the activity of 3′-UTR of *p21* mRNA via some miRNAs. We first sought to identify the potential p21 miRNAs that are regulated by BRD4 in MKN28 cells using target scan algorithms. We search p21 miRNAs using two different target predicting algorithms Target Scan (http://www.targetscan.org) and miRBD (http://mirdb.ord/miRDB), which are miRNA target prediction and functional annotation databases^28,29^. Based on these two algorithms, 20 miRNAs were identified and predicted to target *p21* mRNA (Fig. 5a). Since BRD4 often serves as a positive transcription regulator, we suspected that BRD4-regulated miRNAs would have enhanced expression in gastric cancer cells. Using a miRNA dataset of gastric cancer samples^30^, we identified seven potentially up-regulated p21 miRNAs in gastric cancer cells (Fig. 5a). When we knocked down the expression of *Brd4* and measured the expression levels of these seven miRNAs in MKN28 cells, we found that depletion of *Brd4* down-regulated the expression of miR-106b-5p and miR-519d-3p while having little effect on the other miRNAs (Fig. 5b), indicating that expression of miR-106b and miR-519d-3p is regulated by BRD4. When miR-106b-5p and miR-519d-3p mimics were transfected into MKN28 cells followed by JQ1 treatment, miR-106b-5p but not miR-519d-3p mimics reduced the JQ1-induced cellular levels of p21 (Fig. 5c), indicating that miR-106b targets *p21* mRNA in MKN28 cells. Consistent with this observation, miR-106b-5p inhibitors increased the cellular levels of p21 in MKN28 and SGC-7901 cells (Fig. 5d and Fig. S6). Sequence alignment of miR-106b-5p and the 3′-UTR of *p21* mRNA reveals that there is one complementary binding site of miR-10b-5p within 3′-UTR of *p21* mRNA (Fig. 5d).

Next, we determined whether miR-106b-5p-mediated down-regulation of p21 was sufficient to trigger the senescence. When miR-106b-5p inhibitors were transfected into MKN28 cells, the number of SA-β-Gal-positive cells was significantly increased (Fig. 5e), indicating that inhibition of miR-106b-5p is sufficient to induce cellular senescence. To further investigate whether the down-regulation of miR-106b-5p was responsible for the cellular senescence induced by BRD4 inhibition, we transfected miR-106b-5p mimics into *Brd4* knockdown MKN28 cells, which had increased cellular senescence (Fig. 5f). Rescue of miR-106b-5p expression efficiently suppressed cellular senescence in *Brd4* knockdown cells (Fig. 5f), confirming that cellular senescence in *Brd4* knockdown cells results from the reduced expression of miR-106b-5p and the associated elevated cellular levels of p21.

### BRD4 regulates miR-106b-5p transcription via E2F for cellular senescence

**Fig. 6:**
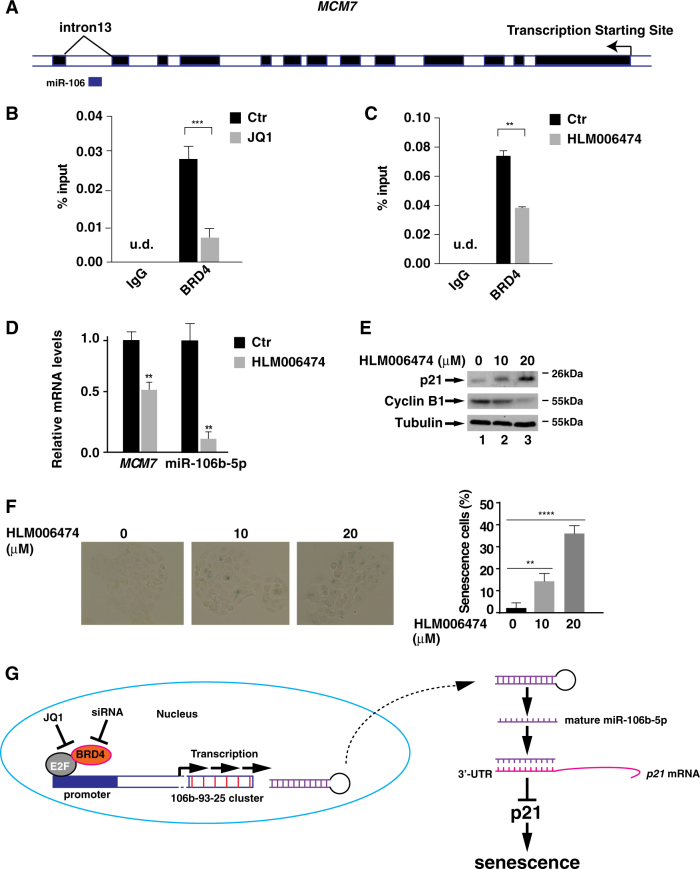
BRD4, together with E2F1, regulates miR-106b-5p transcription and cellular senescence. **a** UCSC genome browser display of *MCM7* gene which hosts miR-106b-5p on *Chr.7* (GRCh38/hg38) assembly. Light box: intron; dark box: exon. **b** and **c** MKN28 cells were treated with either JQ (5 μM) **(B)** or HLM006474 (20 μM) **(C)** for 24 h. ChIP assay was performed using antibodies against IgG or BRD4 and probed for the promoter region of miR-106b-5p. **d** MKN28 cells were treated with HLM006474 (20 μM) for 24 h and the levels of *MCM7* mRNA and mature miR-106b-5p were measured by RT-PCR. **e** MKN28 cells were treated with HLM006474 (10 μM and 20 μM) for 24 h and the levels of p21 and cyclin B1 were measured by immunoblotting. **f** MKN28 cells were treated with indicated concentration of HLM006474 for 4 days and the cells were subject to β-Gal staining. The percentage of β-Gal staining-positive cells is shown on the right. Data represent the average of three independent experiments. **g** Schematic model for the regulation of p21 and cellular senescence by BRD4 in gastric cancer cells. In cancer cells, BRD4 is recruited to the promoter of miR-106b-5p via E2F proteins and activates the expression of miR-106b-5p, which targets the 3′-UTR of *p21* mRNA and suppresses its expression to promote cell proliferation. Inhibition of BRD4 by siRNA or JQ1 results in the down-regulation of miR-106b-5p, leading to the increased expression of p21 and cellular senescence

Having identified that BRD4 regulates cellular senescence via miR-106b-5p/p21 axis, we next assessed how BRD4 regulated the expression of miR-106b-5p. Using the UCSC gene browser^31^, we identified that miR-106b-5p was located in the 13th intron of its host *mini-chromosome maintenance protein 7* (*MCM7)* gene (Fig. 6a), which shares the same promoter as miR-106b-5p^32^. ChIP assay against BRD4 revealed that BRD4 bound to the promoter region of miR-106b-5p in MNK28 cells and treatment of cells with JQ1 alleviated the binding of BRD4 on the promoter (Fig. 6b).

There are three E2F binding sites within the promoter region of *MCM7*, and the expression of MCM7 is regulated by E2F proteins^33^, raising the possibility that BRD4-dependent expression of miR-106b-5p might be regulated by E2F proteins. To test this hypothesis, we examined the binding of BRD4 on the promoter of miR-106b-5p in the presence of E2F inhibitor HLM006474, which inhibits the DNA binding activity of E2F proteins^34^. Treatment of MKN28 cells with HLM006474 efficiently inhibited the binding of BRD4 to the promoter of miR-106b-5p (Fig. 6c), suggesting that E2F regulates the recruitment of BRD4 to the promoter of miR-106b-5p. Importantly, HLM006474 also down-regulated the expression of *MCM7* and the expression of miR-106b-5p (Fig. 6d), suggesting that expression of *MCM7* and miR-106b-5p is regulated by E2F likely from the same promoter. Supporting a role of E2F-mediated expression of miR-106b-5p in cellular senescence, treatment of MKN28 cells with HLM006474 increased the cellular levels of p21 and the number of SA-β-Gal-positive cells (Fig. 6e, f).

## Discussion

Overexpressed *BRD4* is found in a variety of cancers and has been shown to be an adverse predictor for survival in some cancers^26,35,36^. Inhibition of BRD4 and other BET family proteins by BETis has been suggested to be a new strategy for the treatment of cancer^12,37^. In this study, we identified cellular senescence as a mechanism for the anti-proliferative effect of BETi JQ1 in gastric cancer cells. In addition, we demonstrated a BRD4-dependent regulatory pathway via E2F/miR-106b/p21 axis for cellular senescence in gastric cancer cells (Fig. 6g).

In addition to gastric cancer cells, BETis have been shown to induce senescence with increased p21 or p27 in some solid tumors and leukemia^38–40^. However, the detailed mechanism for the BETi-induced p21 expression and the associated cellular senescence reminds unclear. Down-regulation of oncogene c-Myc has been described as a major mechanism responsible for the anti-proliferative effect of BETis in many cancer cells while c-Myc-independent mechanisms also exist^12^. Since c-Myc has been shown to negatively regulate the expression of p21^41^, the up-regulated p21 in JQ1-treated or *Brd4* knockdown MKN28 cells might also result from a down-regulated c-Myc. However, depletion of *Brd4* did not affect the mRNA levels of *c-Myc* (Fig. S7), excluding the possible involvement of c-Myc in the expression of p21 in MKN28 cells. Tumor suppressor p14^ARF^ and p16^INK4A^ have also been shown to be critical regulators of cellular senescence^42,43^. However, p14^ARF^ and p16^INK4A^ don’t seem to be involved in JQ1-induced cellular senescence since JQ1 induced p21 and β-GAL expression in gastric cancer cells regardless of the expression status of p14^ARF^ and/or p16^INK4A^ (Figs. S4&S8).

Regulation of p21 occurs at different levels. At the transcriptional level, p21 can be activated by p53 to induce a permanent cell cycle arrest^16,17^. Nevertheless, p53 is mutated and inactive in MKN28 and SGC-7901 gastric cancer cells (Table 1)^44,45^, where the levels of p21 was dramatically up-regulated with JQ1 treatment or *Brd4* depletion (Figs. 2 and 3). JQ1 also enhanced p21 expression in WT p53 expressing MKN45 and AGS cells without affecting p53 levels (Table 1) (Fig. S8). These data indicate a p53-independent but BRD4-dependent regulation of p21. Consistent with this notion, we observed that p21 expression was largely controlled at the post-transcriptional level by miRNAs. Overexpression of miR-106b-5p down-regulated the cellular levels of p21, whereas inhibition of miR-106b-5p up-regulated the levels of p21 in MKN28 and SGC-7901 cells (Fig. 5d and Fig. S6). In addition, inhibition of miR-106b-5p was sufficient to induce senescence and overexpression of miR-106b-5p blocked cell senescence in BRD4-knockdown cells (Figs. 5e, f). Therefore, BRD4 indirectly modulates the levels of p21 and cell senescence by regulating the expression of miR-106b-5p. It has to be noted that p21 siRNA partially reversed JQ1-induced cellular senescence (Fig. 3d) and the senescence induced by miR-106b-5p inhibitor (Fig. 5e) was not as dramatic as senescence induced by 5 μM JQ1 (Fig. 2c). These data indicate that BRD4 might regulate cellular senescence with additional unidentified mechanisms.

**Table 1:** TP53, p14^ARF^ and p16^ink4a^ gene alterations in gastric cancer cell lines

Genes	*TP53* ^*(ref)*^	***p16*** ^***ink4a(ref)***^	***p14*** ^***ARF(ref)***^
Cell Lines			
AGS	_Wild-type_^57^	_ + _^58,59^	− ^60^
AZ521	_Mutation (S303N)_^61^	NA	NA
MKN28	_Mutation (I251L)_^56,62^	− ^59,62^	− ^63^
MKN45	_Wild-type_^57,62^	− ^62^_/ + _^59^	− ^63^
SGC-7901	_Mutation (E204A)_^64^	_ + _^65^	_ + _^65^
NCI-N87	_Mutation (R248Q)_^66^	− ^59^	NA
SNU-16	_Mutation (Y205F)_^66^	− ^59^	NA
SUN-5	_Del (Codon262-269)_^(66)^	NA	NA

+ represents normal or over-expression detected by western blot. − represents under-expression detected by western blot

How does BRD4 regulate the transcription of miR-106b-5p? miR-106b-5p is located in the intron 13 of the host *MCM7* gene (Fig. 6a) and its transcription is under control by the same regulatory unit as *MCM7*^32^. The expression *MCM7* is largely regulated by the E2F proteins^33^. It apears that the expression of miR-106b-5p is also regulated by E2F since inhibition of E2F by HLM006474 diminished the binding of BRD4 to the promoter of miR-106b-5p and its expression (Fig. 6c). BRD4 has been shown to associate with acetylated E2F1 to regulate the expression of certain genes^46^. A similar mechanism might be utilized to regulate the expression of miR-106b-5p. BRD4 could be recruited to the promoter by its association with E2F via its two bromodomains and facilitates the transcription of miR-106b-5p (Fig. 6g). MCM7 plays an essential role in the G1/S phase transition^47^. The G1/S cell cycle arrest in JQ1-treated MKN28 cells (Fig. 2) might result from the concomitant reduced expression of *MCM7* (Fig. S9). As such, E2F-dependent recruitment of BRD4 to the promoter of *MCM7* and miR-106b-9p might have dual effects on the cell proliferation. While the BRD4-dependent expression of *MCM7* modulates the G1/S transition, BRD4-dependent expression of miR-106b-5p, which targets the 3′-UTR of *p21* mRNA, regulates cellular senescence.

miR-106b-5p is known to be an oncogenic miRNA in a variety of cancers, including gastric cancer, hepatocellular carcinoma, and breast cancer^48–50^. Interestingly, BRD4 has also been found to have tumor-promoting activity in these cancers^51–53^. The oncogenic activity of BRD4 might partially derive from its ability to up-regulate the expression of miR-106b-5p. BRD4 is overexpressed in different cancers, including gastric cancer (Fig. 1)^26,35,36^. Interestingly, miR-106b-5p is also overexpressed in gastric cancer patient samples (Fig. S10). It would be interesting to determine whether miR-106b-5p accounts for the tumor-promoting activity of BRD4 in gastric cancer and other cancers. Since miRNAs often have multiple targets, it is possible that BRD4-mediated miR-106b-5p might have additional functions in gastric cancer cells. For example, miR-106b-5p has been shown to be involved in TGF-β-dependent cell cycle arrest and apoptosis in gastric cancer cells^50^.

Cellular senescence is triggered in response to diverse forms of cellular stress, including activation of oncogene and DNA damage^14^. While it is clear that inhibition of BRD4 induces cellular senescence in some gastric cancer cells and the senescence partially accounts for the halted proliferation of these gastric cancer cells (Fig. 1), it has to be noted that BRD4 has been shown to be essential for the senescence-associated secretory phenotype (SASP) in oncogene- or DNA damage-induced cellular senescence^54^. The reduced SASP response compromised the immune surveillance to remove senescent cells^54^. Different from oncogene-induced senescence, BRD4 inhibition-induced senescence of gastric cancer cells was not associated with increased SASP factors since the expression of SASP factors, including IL1A, IL1B, and IL8 was down-regulated in BRD4-inhibited MKN28 cells (data not shown). It remains to be determined whether the reduced expression of SASP factors in gastric cancer cells would contribute to the suppression of immune surveillance in vivo and affect the efficacy of the BETis in the treatment of gastric cancer.

All together, our studies have explored the anti-proliferative effect of JQ1 in gastric cancer cells and also identified BRD4-dependent regulation of cellular senescence via E2F/miRNA-106b-5p/p21 axis as an underlying mechanism. These studies not only provide new insights into the BRD4-mediated cancer cell proliferation but also provide potential new targets for the treatment of gastric cancer by targeting the E2F/miRNA-106b-5p/p21 axis.

## Materials and methods

### Cell lines, reagents, plasmids, and antibodies

Human gastric cancer cell lines AGS, AZ521, NCI-N87, MKN28, MNK45, SGC-7901, SNU-5, SNU-16 were maintained in RPMI-1640 medium supplemented with 10% FBS. JQ1 has been described previously^11^. The p53, INK4A and ARF expression status of these gastric cancer cells is listed in Table 1. E2F inhibitor HLM006474 was purchased from Tocris Bioscience. Lipofectamine® RNAiMAX Transfection Reagent and siRNAs targeting *Brd4* or *p21* were from ThermoFisher. Antibodies against p21, Cyclin B1, p14^ARF^, p53 and Tubulin were from Santa Cruz Biotechnology; p16^INK4A^ antibody was from Abcam. BRD2 and PARP antibodies were from Cell Signaling Technology; BRD3 and BRD4 antibodies were from Bethyl Laboratories. Lentiviral shBRD2 plasmid is a generous gift from Dr. Frank. Lentiviral shBRD3 and shBRD4 plasmids are purchased from Sigma. *p21* 3′-UTR luciferase plasmid is a kind gift from Dr. He.

### Patient samples and immunohistochemical staining

All 104 gastric tissue samples, including 52 gastric cancer samples and 52 paired normal gastric mucosa samples, were collected between October 2014 to June 2016 from Fujian Provincial Cancer Hospital. The study was approved by the Ethics Committee of Fujian Provincial Cancer Hospital.

Immunohistochemical staining for BRD4 has been described previously (23). BRD4 staining intensity was graded as previously described with a score 0 to 3. Samples with a score 0 were graded as negative, samples with a score 1–3 were graded as positive (weak (1), moderate (2), and strong (3)).

### Proliferation and soft agar assay

Cell proliferation and soft agar assays have been previously described^55^.

### Invasion and migration assay

Cell invasion assay was performed using Transwell invasion chambers (Becton, Dickinson, and Company) according to manufacturer’s instructions. For migration assay, cells were grown to nearly 100% confluency and serum starved overnight before producing the scratch wound by dragging a 200 μL pipette tip across the layer. Detached cells were washed away with cell culture medium. Cells were cultured in RPMI with either dimethyl sulfoxide (DMSO) or JQ1 for the indicated time. The closure of the wound was monitored by microscopy at the indicated time points after inflicting the wound.

### β-gal staining assay

β-gal staining was performed using Senescence β-Galactosidase Staining Kit (Cell Signaling Technology). Briefly, 5000 cells were seeded and cultured with DMSO or JQ1 in 6 well plates. After 4 days, cells were incubated with staining solution overnight. Senescence β-Gal staining pictures were taken 16 h later using EVOS XL Core Microscope (Life Technologies).

### Chromatin immunoprecipitation and quantitative real-time PCR

The chromatin immunoprecipitation (ChIP) assay was performed as described previously^56^. MKN28 cells RNA was extracted using Aurum™ Total RNA Mini Kit (BIO-RAD). Complementary DNA was synthesized with an iScript™ cDNA Synthesis Kit (BIO-RAD). Quantitative real-time PCR was performed using a BIO-RAD SYBR Green PCR kit with a 7300 real-time PCR system (ABI). PCR primers for various target genes were synthesized by integrated DNA technologies (IDT). Samples were normalized using the housekeeping gene GAPDH.

### MicroRNA quantification, siRNAs, and MicroRNA mimics transfection

Mature microRNA quantification was performed using QuantiMir™ RT Kit from System Biosciences (SBI). All microRNA quantitative real-time PCR primers were synthesized from IDT according to QuantiMir™ RT Kit instruction. MicroRNA mimics and inhibitors were purchased from GE Dharmacon. MicroRNA mimics and siRNAs transfection was performed using Lipofectamine® RNAiMAX Transfection Reagent.

### Generation of BRD2, BRD3, and BRD4 shRNA in MKN28 Cells with lentivirus particles

HEK293T cells were transfected with packaging plasmids VSV-G, Gag-Pol, and lentiviral vectors that harbor shBRD2, shBRD3, and shBRD4, respectively. After 48 h, culture medium containing viral particles was collected and passed through 0.22 μm syringe filters. MKN28 cells were infected with viral particles for 2 days with the addition of polybrene at a concentration of 8 μg/ml. Cells were selected with puromycin for 2 days before experiments.

### Clonogenic assay

MKN28 cells were infected with lentiviruses expressing shCtr, shBRD2, shBRD3, and shBRD4, respectively and selected with puromycin for 2 days. Cells were then seeded (1 × 10^4^ cells/well) in 6-well plate and kept growing for another 4 days. Cells were fixed with glutaraldehyde (6.0% v/v) and stained with 0.5% crystal violet for 20 min at room temperature followed by washing with water for 4 times. The plates were dried at room temperature overnight. Pictures were taken using Chemidoc Imaging System (BioRad).

### Luciferase reporter assay

MKN28 cells were transfected using Lipofectamine® with various plasmids and luciferase reporters. Firefly and Renilla luciferase activities were measured with the Dual-Luciferase assay system from Promega.

### Statistical analysis

All data are presented as mean ± SD unless otherwise stated. Student unpaired t-test was used to analyze the data. Statistical significance was determined using GraphPad Prism6 software (GraphPad). For all data presented, *P* value ≤ 0.05 was considered statistically significant. (*P ≤ 0.05, **P ≤ 0.01, ***P ≤ 0.005, **** *P* ≤ 0.001).

## Electronic supplementary material

Supplementary Figures

Supplementary Figures
