# Supplementary figures and images for: BRD4 regulates cellular senescence in gastric cancer cells via E2F/miR-106b/p21 axis
Source: Cell Death Dis. 2018 Feb 12;9(2):203. doi: 10.1038/s41419-017-0181-6 (PMC5833665; doi:10.1038/s41419-017-0181-6)

S1

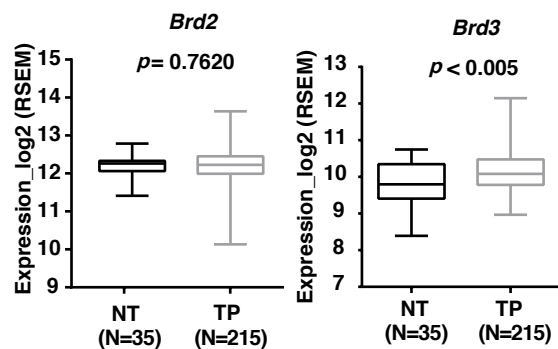

S2

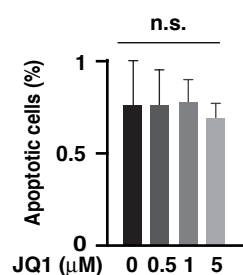

S3

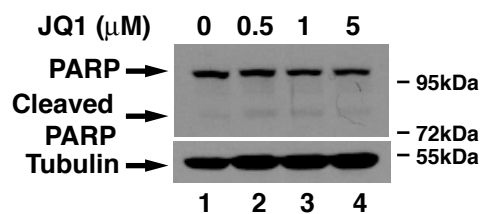

S4

SGC-7901

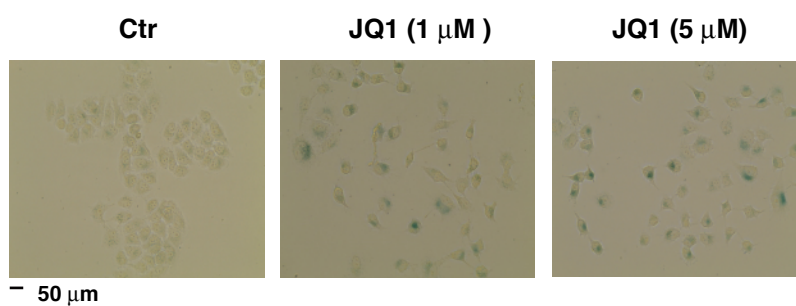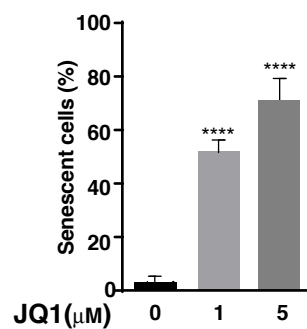

AGS

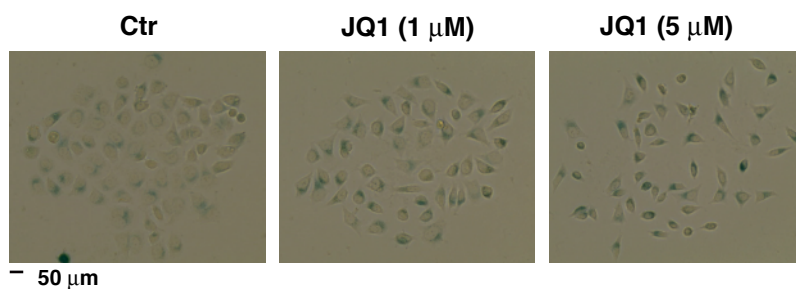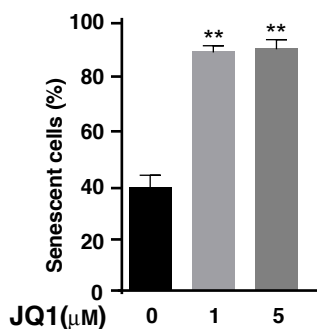

MKN45

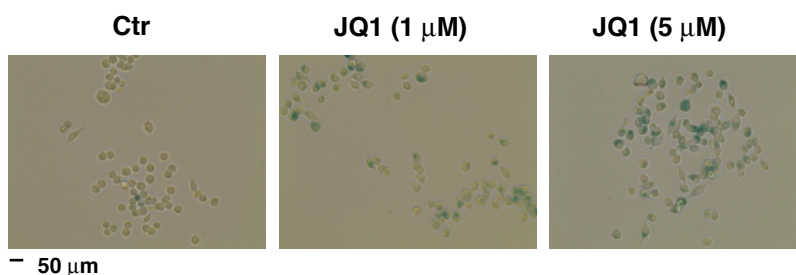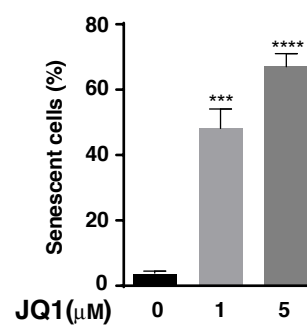

S5

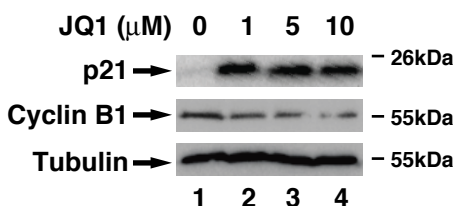

S6

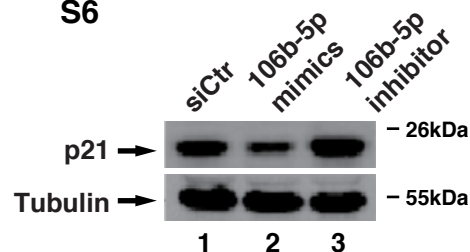

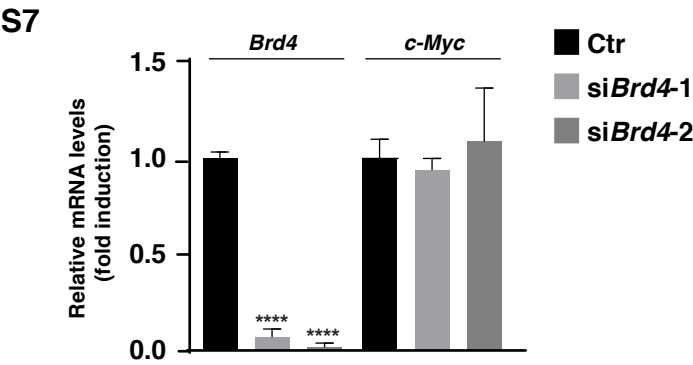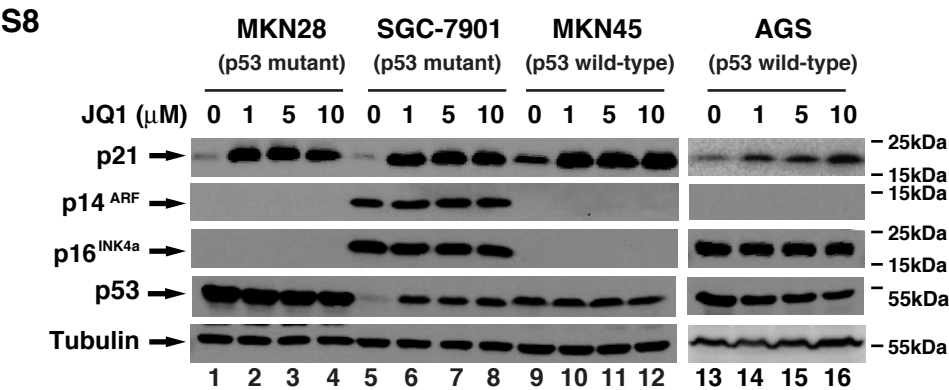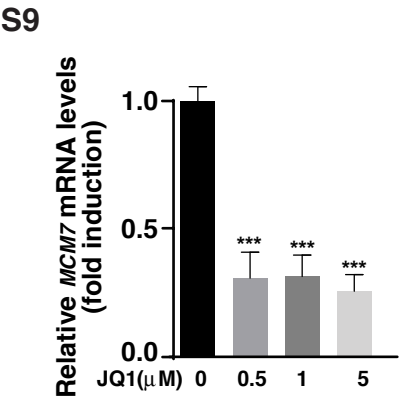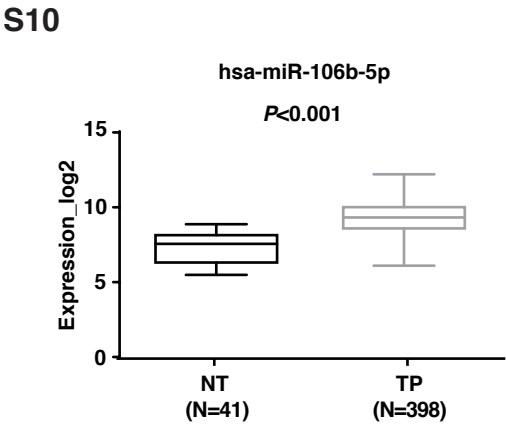

Supplement: Supplementary file 1 — Supplementary Figures [file 41419_2017_181_MOESM1_ESM.pdf]
