# Supplementary material for: BRD4 regulates cellular senescence in gastric cancer cells via E2F/miR-106b/p21 axis
Source: Cell Death Dis. 2018 Feb 12;9(2):203. doi: 10.1038/s41419-017-0181-6 (PMC5833665; doi:10.1038/s41419-017-0181-6)
Supplement: Supplementary file 2 — Supplementary Figures [file 41419_2017_181_MOESM2_ESM.docx]

**Supplementary Figure legends**

**Fig. S1.** BRD2 and BRD3 mRNA levels in NT (patient number = 35) versus TP (patient number = 215) are acquired through TCGA Firebrowse portal (<http://firebrowse.org/>). Box-plots of BRD2 and BRD3 mRNA levels in NT vs TP are visualized using Graphpad Prism and p-value is calculated and displayed.

**Fig. S2**. JQ1 does not induce apoptosis of MKN28 cell. Annexin V and PI staining of MKN28 cells treated with DMSO or various concentration of JQ1 for 24 h. The percentage of cells is indicated. The percentage of apoptotic cells from three independent experiments ± SD is shown on the right.

**Fig. S3**. MKN28 cells were treated with indicated concentration of JQ1 for 24 h. Cell lysates were immunoblotted for the full-length and the cleaved PARP using anti-PARP antibody.

**Fig. S4.** SGC-7901, AGS, and MKN45 cells were treated with indicated concentration of JQ1 for 72 h, and cellular senescence was measured using Senescence β-Galactosidase Staining Kit (Cell Signaling). The percentage of β-Gal staining-positive cells is shown in the right. Data represent the average of three independent experiments.

**Fig. S5.** SGC-7901 cells were treated with different concentrations of JQ1 for 24 h, and the cell lysates were immunoblotted for indicated proteins.

**Fig. S6.** miR-106b-5p mimics and inhibitors were transfected into SGC-7901 cells. Forty-eight hours later, the levels of indicated proteins were measured by immunoblotting.

**Fig. S7.** MKN28 cells were transfected with control or Brd4 siRNA for 48 h. The levels of *Brd4* and *c-Myc* mRNA were measured by RT-PCR.

**Fig. S8.** MKN28, SGC-7901, MKN45, and AGS cells were treated with DMSO or various concentration of JQ1 for 24 h, and cell lysates were immunoblotted for indicated proteins.

**Fig. S9.** MKN28 cells were treated with different concentration of JQ1 for 24 h, and the levels of *MCM7* mRNA were measured by RT-PCR.

**Fig. S10** Visualization of mature miR-106b-5p level in NT vs. TP utilizing Firebrowse portal through TCGA dataset.
